# Time-Course Gene Set Analysis for Longitudinal Gene Expression Data

**DOI:** 10.1371/journal.pcbi.1004310

**Published:** 2015-06-25

**Authors:** Boris P. Hejblum, Jason Skinner, Rodolphe Thiébaut

**Affiliations:** 1 Univ. Bordeaux, ISPED, Centre INSERM U897-Epidemiologie-Biostatistique, F-33000 Bordeaux, France; 2 INSERM, ISPED, Centre INSERM U897-Epidemiologie-Biostatistique, F-33000 Bordeaux, France; 3 INRIA, Team SISTM, F-33000 Bordeaux, France; 4 Vaccine Research Institute-VRI, Hôpital Henri Mondor, Créteil, France; 5 Baylor Institute for Immunology Research, Dallas, Texas, United States of America; University of Toronto, CANADA

## Abstract

Gene set analysis methods, which consider predefined groups of genes in the analysis of genomic data, have been successfully applied for analyzing gene expression data in cross-sectional studies. The time-course gene set analysis (TcGSA) introduced here is an extension of gene set analysis to longitudinal data. The proposed method relies on random effects modeling with maximum likelihood estimates. It allows to use all available repeated measurements while dealing with unbalanced data due to missing at random (MAR) measurements. TcGSA is a hypothesis driven method that identifies a priori defined gene sets with significant expression variations over time, taking into account the potential heterogeneity of expression within gene sets. When biological conditions are compared, the method indicates if the time patterns of gene sets significantly differ according to these conditions. The interest of the method is illustrated by its application to two real life datasets: an HIV therapeutic vaccine trial (DALIA-1 trial), and data from a recent study on influenza and pneumococcal vaccines. In the DALIA-1 trial TcGSA revealed a significant change in gene expression over time within 69 gene sets during vaccination, while a standard univariate individual gene analysis corrected for multiple testing as well as a standard a Gene Set Enrichment Analysis (GSEA) for time series both failed to detect any significant pattern change over time. When applied to the second illustrative data set, TcGSA allowed the identification of 4 gene sets finally found to be linked with the influenza vaccine too although they were found to be associated to the pneumococcal vaccine only in previous analyses. In our simulation study TcGSA exhibits good statistical properties, and an increased power compared to other approaches for analyzing time-course expression patterns of gene sets. The method is made available for the community through an R package.

## Author Summary

Gene set analysis methods use prior biological knowledge to analyze gene expression data. This prior knowledge takes the form of predefined groups of genes, linked through their biological function. Gene set analysis methods have been successfully applied in transversal studies, their results being more sensitive and interpretable than those of methods investigating genomic data one gene at a time. The time-course gene set analysis (TcGSA) introduced here is an extension of such gene set analysis to longitudinal data. This method identifies *a priori* defined groups of genes whose expression is not stable over time, taking into account the potential heterogeneity between patients and between genes. When biological conditions are compared, it identifies the gene sets that have different expression dynamics according to these conditions. Data from 2 studies are analyzed: data from an HIV therapeutic vaccine trial, and data from a recent study on influenza and pneumococcal vaccines. In both cases, TcGSA provided new insights compared to standard approaches thanks to an increased sensitivity compared to other approaches. Those results highlight the benefits of the TcGSA method for analyzing gene expression dynamics.

This is a *PLOS Computational Biology* Methods paper.

## Introduction

Microarray experiments are increasingly used for evaluating changes in gene expression over time. The analysis of the temporal change of gene expression should help in understanding the complex mechanisms of gene regulation. For instance, transcriptional profiles have been repeatedly measured to study the change in gene expression during the natural history of SIV/HIV infection [1, 2] or to evaluate the effect of vaccines [3, 4]. In the applications considered in this paper [5, 6], the investigators wanted to detect the genes for which the abundance changed over time after a vaccination (against HIV, influenza or pneumococcus).

In order to analyze such longitudinal high-dimensional data, several approaches have been suggested including a gene-by-gene statistical analysis [7, 8], dimension reduction methods [9] or gene set analysis [10]. A gene set is a group of genes that are *a priori* co-regulated or functionally linked. Examples of such gene set relating to biological processes or pathways are those defined by KEGG [11], Gene Ontology [12] or Chaussabel’s functional modules [13]. The gene set analysis [14–16] is supposed to be more powerful than a gene-by-gene analysis because it can detect a change of expression of a group of genes although none of them show a very high absolute fold change. Furthermore, a change of all genes in a given pathway may be biologically more meaningful than a large increase of a single gene. Also, provided that the gene sets are well defined, the result should be more sound and comparable across studies than a gene-by-gene analysis [14]. Finally, gene set analysis avoids a second step for a global interpretation as described in the “bottom up” approach [10, 17].

**Fig 1:**
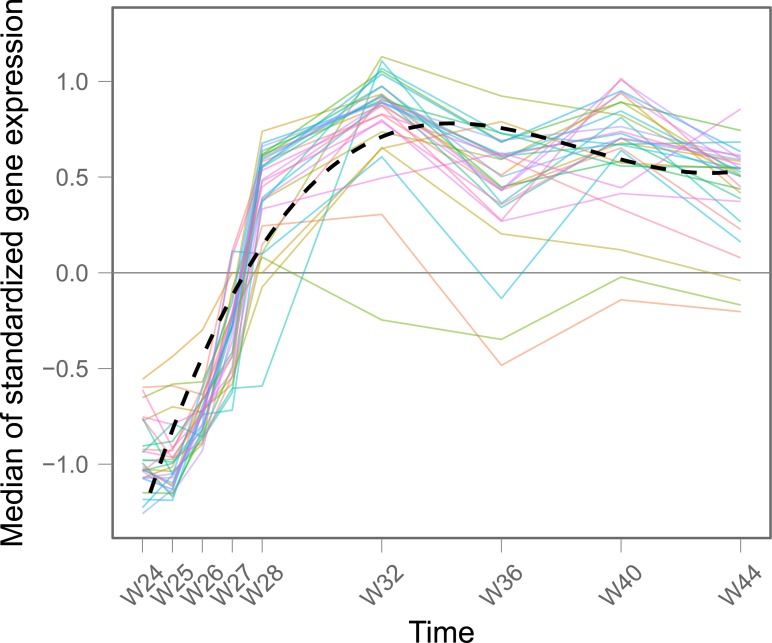
Example of a homogeneous gene set (M1.2: interferon—from the DALIA trial, after treatment interruption). Each line is the median expression of a gene inside this particular gene set across all the patients. The expression of the genes inside this gene set is quite homogeneous and it is easy to identify a global time trend, displayed by the dashed black line (smoothed median). For more information see the presentation of the DALIA-trial in the “Results” section.

**Fig 2:**
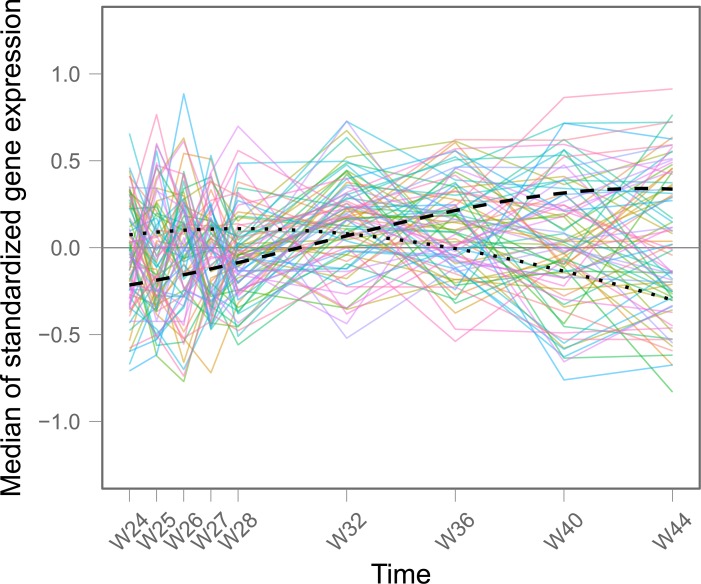
Example of a heterogeneous gene set (M4.16: cell cycle—from the DALIA trial, after treatment interruption). Each line is the median expression of a gene inside this particular gene set across all the patients. The expression of the genes inside this gene set is rather heterogeneous. This makes it difficult to identify any time trend, as the mean expression inside this gene set stays close to zero. However a closer look reveals two distinct time trends, displayed by the two respectively dashed and dotted black lines (smoothed medians). For more information see the presentation of the DALIA-trial in the “Results” section.

The analysis of longitudinal microarray experiments through a gene set approach is not trivial because the dynamics of gene expressions inside a gene set can be complex and heterogeneous. This has already been underlined in some of the approaches developed to analyze gene sets [15, 18–20]. Fig 1 shows an example of a homogeneous gene set, whereas Fig 2 shows an example of a heterogeneous one. Actually, such a heterogeneity is frequently observed [20], and cannot be ignored, as genes inside a functional gene set are not expected to change their expression synchronously. Moreover this heterogeneity can be biologically meaningful by itself. Prieto et al. [21] provide an example from a cancer application, where deregulated pathways are of primary biological interest. They identified heterogeneous gene sets linked to acute promyelocytic leukemia. Another example is given by Hu et al.: pathways affected by the HER2, such as the KEGG pathways of ‘Ubiquitin mediated proteolysis’, ‘Glioma’, and ‘Prostate cancer’ were identified by studying heterogeneity [22]. The main advantage of detecting the heterogeneity inside a gene set is to detect any change over time whatever the specification of the model for the trends. In other words, the dynamics of gene expression inside a stable gene set will be summarized by a flat slope and no heterogeneity. Hence, in the spirit of [19], to find any significant change of the overall expression of genes inside a gene set over time, we suggest to look for any significant trend over time or any heterogeneity between gene trends inside the gene set.

Several approaches have already been proposed to analyze longitudinal measurements of gene expression [7, 23–28], but only a few include gene set analysis [10, 18, 29–31]. Among the latter, all but Nueda et al. [18] fail to account for possible heterogeneity inside a gene set. An extension of the popular Gene Set Enrichment Analysis (GSEA) method [14] is available for the analysis of time series data: GSEA for time series. Unfortunately, it does not account for the structure of longitudinal data, simply treating all observations as independent and calculating Pearson correlation of the genes with the time variable. Therefore this is quite a limited modeling strategy for longitudinal data. The globalANCOVA procedure developed by Hummel et al. [29] focuses on the comparison of groups, testing whether there is a group influence on change over time of any gene expression inside a gene set. In practice, the global null hypothesis tested is quite flexible relying on the ANOVA framework, but cannot accommodate missing values. Wang et al. [10] proposed to use a linear mixed effects model to explain gene expression inside a gene set. They considered a random effect for the array level rather than for the patient or the gene level. Zhang et al. [30] proposed a robust non-parametric approach to compare gene expression dynamics between different treatment-groups. Wu et al. [31] proposed the CAMERA procedure, a gene set test based on linear modeling that takes into account inter-gene correlations. However, CAMERA accounts for neither potential heterogeneity inside a gene set nor for repeated measurement correlation. Of note, it is not possible to look at the change of gene expression in only one group using Zhang et al. [30] approach. In contrast, the PCA-maSigFun procedure developed by Nueda et al. [18, 32] can account for possible heterogeneity inside a gene set. It is based on a Principal Component Analysis (PCA) of each gene set followed by a linear regression of the significant principal components (i.e. components that have a variation above the mean gene variance) over time. However, they did not consider time-course experiments where repeated measures are available for multiple patient.

Gene set analysis methods can also be distinguished by their choice of the null hypothesis. Those can be classified into two main types of hypothesis: i) the competitive null hypothesis, that tests the genes inside a given gene set against all the other genes outside the gene set; ii) the self-contained null hypothesis, that only uses the genes inside the gene set of interest [20, 33, 34]. In the present paper, interest is focused on self-contained null hypotheses because the question was “Which gene sets have a change of gene abundance over time?”. According to Emmert-Streib et al. [35], self-contained gene set tests are biologically easier to interpret and can be more powerful compared to competitive tests. Self-contained gene set tests are especially appropriate in a hypothesis driven context where *a priori* defined gene sets are validated and relevant regarding the biological question.

We propose the implementation of a hypothesis driven method that directly tests the time-course significance of predefined gene sets: the *Time-course Gene Set Analysis* (TcGSA). It relies on the use of linear mixed effect models, a very useful and well established statistical tool [36, 37] especially suited for longitudinal settings. By using all available repeated measures, it provides increased statistical power. TcGSA can accommodate for heterogeneity of gene expression within the gene sets through random effects, and is robust to unbalanced designed due to missing (at random) values thanks to the maximum likelihood estimates. No previously proposed approach combines all of TcGSA features. A simulation study demonstrated the good statistical performance of the proposed method. It has been applied to two studies: one HIV vaccine trial, and one influenza and pneumoccocal vaccine study [6], using the same definition of gene sets [13] that is increasingly used in systems immunology research [38–42]. Compared to gene-by-gene analyses, TcGSA disclosed changes of additional gene sets that endorse previous conclusions [6], but also revealed common pathways across the three vaccines.

## Methods

### Time-course gene set analysis

The TcGSA method includes three steps: 1) modeling gene expression in a gene set with mixed models, 2) testing the significance of a gene set, and 3) estimating individual gene profiles.

#### 1. Modeling gene expression in a gene set with mixed models

Let *S* be a gene set of interest. We start by the case of a one group experiment, where each patient act as her/his own respective control, her/his condition changing over time. The expression of genes inside *S* is modeled over time according to a function *f* as:

for all the genes *g* ∈ *S*,

$$\begin{array}{c} y_{gpi}=\mu +\beta_{g}+c_{gp}+f_{g}\left(t_{i}\right)+\varepsilon_{gpi} \end{array}$$ (1)

where *y*_*gpi*_ is expression of the *g* ^*th*^ gene for the *p* ^*th*^ patient at the *i* ^*th*^ time, *μ* is the intercept in the gene set *S*, *β*_*g*_ is the fixed effect of the *g* ^*th*^ gene, *c*_*gp*_ ∼ 𝓝(0, *σ*_*c*_) is a random effect grouped by the *g* ^*th*^ gene of the *p* ^*th*^ patient, *t*_*i*_ is the *i* ^*th*^ measurement time, *ɛ*_*gpi*_ ∼ 𝓝(0, *σ*) is an error term. Finally *f*_*g*_(*t*_*i*_) is a function of time, that can be linear, polynomials, etc. Every time coefficient of the trend *f*_*g*_(*t*_*i*_) is actually divided into a fixed effect *η*_⋅_ (representing the average trend in the gene set *S*) and a random effect *h*_*g*,⋅_ ∼ 𝓝(0, *σ*_*h*_⋅__) grouped on the gene *g*, accounting for the possible heterogeneity between the genes in the gene set *S*. In this paper we focus on three forms for *f*_*g*_ (but other forms, such as exponential, etc. could easily be envisaged):

- linear polynomials:
$f_{g}(t)=\left(\eta_{1}+h_{g,1}\right)t$
- cubic polynomials:
*f*_*g*_(*t*) = (*η*_1_+*h*_*g*,1_)*t*+(*η*_2_+*h*_*g*,2_)*t* ^2^+(*η*_3_+*h*_*g*,3_)*t* ^3^
- natural cubic splines:
$f_{g}(t)=\sum_{k=1}^{K+1}\left(\eta_{k}+h_{g,k}\right)N_{k}(t)$

where the *N*_*k*_(*t*) form the natural cubic splines basis [43] for the time variable *t* (with *K* internal knots), *η*_⋅_ are the fixed effects of time shared across the gene set *S*, and *h*_*g*,⋅_ are the random effects of time accounting for possible heterogeneity between genes. (*h*_*g*,1_,…, *h*_*g*, *d*_) ∼ 𝓝(0,Σ_*h*_) with *d* being the degree of the time function, and for *k* = 1,…, *d* *h*_*g*, *k*_ ∼ 𝓝(0, *σ*_*h*_*k*__). Alternatively, one can make the assumption that the patient effect is the same for all the genes. In that case, the random effect *c* is no longer grouped on the gene level, and the model can be written as:

for all the genes *g* ∈ *S*,

$$\begin{array}{c} y_{gpi}=\mu +\beta_{g}^{{}^{\prime }}+c_{p}^{{}^{\prime }}+f_{g}\left(t_{i}\right)+\varepsilon_{gpi} \end{array}$$ (1bis)

with $c_{p}^{\prime }∼𝓝(0,\sigma_{c^{\prime }})$ the random effect of the patient *p*, and $\beta_{g}^{\prime }∼𝓝(0,\sigma_{\beta^{\prime }})$ the random effect of the gene *g*. This alternative modeling has the advantage to be more parsimonious than the Eq (1), with less parameters to be estimated.

Let’s now consider the case of a multiple group experiment (such as treatment/vaccine groups for instance). The expression of genes inside *S* is modeled over time according to a function *f*_*g*, *m*_ that is now stratified on the groups:

for all the genes *g* ∈ *S*,

$$\begin{array}{c} y_{mgpi}=\mu +\beta_{g}+\delta_{m}+c_{gp}+f_{g,m}\left(t_{i}\right)+\varepsilon_{gpi} \end{array}$$ (2)

where *m* indicates which group is concerned and *δ*_*m*_ is the fixed intercept of the *m* ^*th*^ group, everything else being the same as in the Eq (1).

#### 2. Testing the significance of a gene set

In TcGSA, a “significant” gene set is a gene set whose expression is not stable either over time (in one group experiments) or over groups (in several groups experiments), once between genes and patients variability is taken into account. In other words, we want to test the significance of the time trend while being sensitive to both homogeneous and heterogeneous changes of gene expression over time inside a gene set. Testing the significance of a given gene set *S* therefore means testing both fixed and random effects at once, in a single test. A likelihood ratio test is the natural way to do so, fitting models under both the null hypothesis and the alternative.

In the case of one group experiment (Eqs (1) and (1bis)) the null hypothesis (H_0_) is that the genes inside *S* are stable over time, *i.e.* that their expressions are constant and homogeneous over time (all coefficients of the function of time *f* are not significantly different from zero). The alternative hypothesis (H_1_) is that the genes inside *S* vary significantly over time:

$$\begin{array}{cc} {\text{(H}}_{\text{0}}\text{):}\;\; & \forall \;k,\;\eta_{k}=0\;\text{and}\;\sigma_{h_{k}}=0 \end{array}$$ (1.0)

$$\begin{array}{cc} {\text{(H}}_{\text{1}}\text{):}\;\; & \exists \;k,\;\eta_{k}\neq 0\;\;\text{or}\;\;\sigma_{h_{k}}\neq 0 \end{array}$$ (1.1)

In the case of a multiple group experiment (Eq (2)), the null hypothesis is that inside the gene set *S*, the evolution of gene expressions over time is the same regardless of the group. The alternative hypothesis is that time trends *f* are different depending on the group *m*:

$$\begin{array}{cc} {\text{(H}}_{\text{0}}\text{):}\;\; & \forall \;m,\;\;f_{g,m}\left(\cdot \right)=f_{g}\left(\cdot \right) \end{array}$$ (2.0)

$$\begin{array}{cc} {\text{(H}}_{\text{1}}\text{):}\;\; & \exists \;m,\;m^{\prime }\;\;\text{such}\;\text{that}\;\;\;\;f_{g,m}\left(\cdot \right)\neq f_{g,m^{\prime }}\left(\cdot \right) \end{array}$$ (2.1)

In both case, one model is fitted under the null hypothesis, and one is fitted under the alternative. The likelihood ratio is then computed.

However, since both fixed and random effects are tested simultaneously in this likelihood ratio, its null distribution is not the standard chi-square distribution (because of boundary constraints due to the variance of random effects). According to Self et al. [44], it can be approximated by a mixture of chi-square distributions with the following formula:

LRH0∼∑k=qq+r(rk-q)2-rχ(k)2

where *q* is the number of fixed effects and *r* the number of random effects to be tested simultaneously. This approximation implies that the tested random effects are independent of one another [45–47]. This seems an acceptable assumption according to our simulations under the null (see S1 and S2 Figs). This allows to compute a p-value for the significance of the variation of a given gene set over time.

When several gene sets are investigated at a time, it is necessary to take into account multiple testing. A number of procedures are available to do so [48]. As the TcGSA is mostly an exploratory analysis (even though hypothesis driven in the sense that gene set are defined *a priori*), we recommend using the Benjamini-Yekutieli procedure for controlling the False Discovery Rate [49], as gene sets are necessarily correlated between each others and this procedure is robust to some of these dependances. Other mutliple testing correction procedures are available in the *TcGSA* R package.

#### 3. Estimation of individual gene profiles

In the estimation of linear mixed model, it is common to use the Restricted Maximum Likelihood (REML) instead of the classic Maximum Likelihood (ML) in order to avoid biased estimates of the variance components [50]. But note that REML cannot be used to estimate the likelihood ratios presented here. Indeed, REML estimation of the likelihood ratio between two models can only be used when both models have the same fixed part [51]. Since here the compared models (under H_0_ and under H_1_) have different fixed components (due to the *η*_⋅_ coefficients under H_1_), the use of ML estimation is needed.

**Fig 3:**
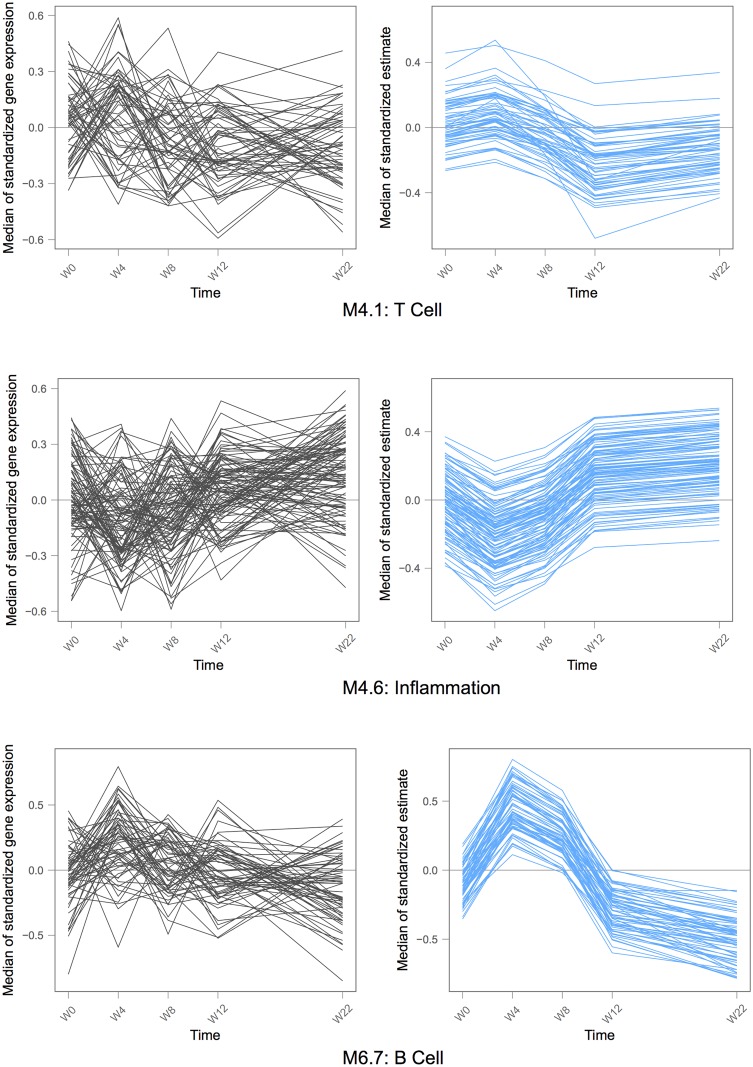
Three significant gene sets during pre-ATI in DALIA-1. Each line is the median over the patients of the expression of one gene. Each graph shows all the genes in one particular gene set. The left graph displays the raw gene expression, the right one displays the estimations from the mixed model for the same gene set. The expressions have been centered and reduced for this representation.

**Fig 4:**
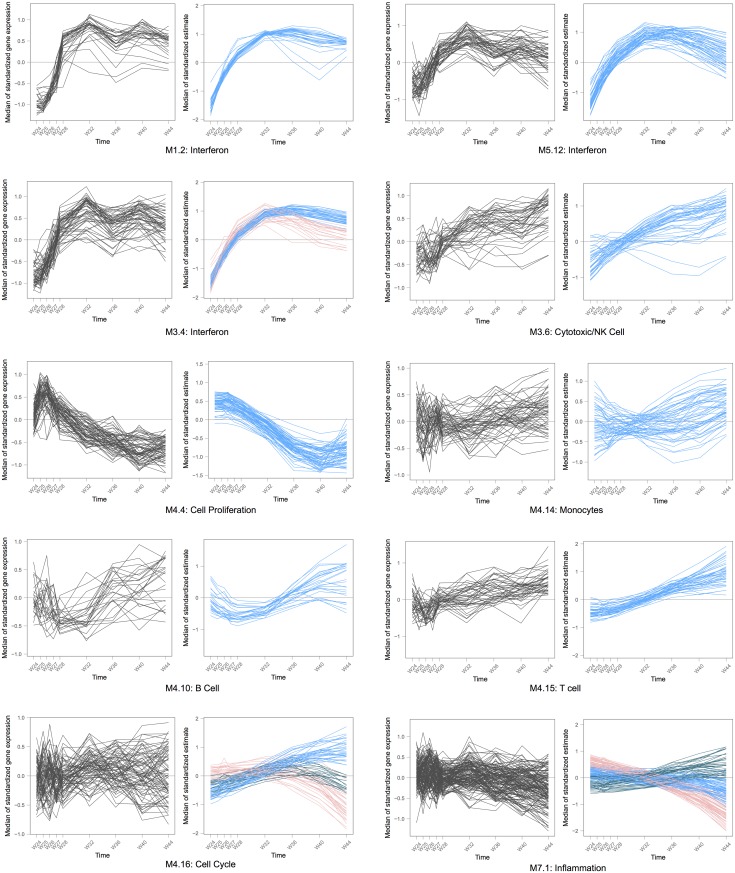
Ten significant gene sets during post-ATI in DALIA-1. Each line is the median over the patients of the expression of one gene. Each graph shows all the genes in one particular gene set. The left graph displays the raw gene expression, the right one displays the estimations from the mixed model for the same gene set. If several dynamics are identified by the gap statistics among the estimated expressions inside one gene set, they are displayed in different colors—such as for the gene sets M 4.16 and M 7.1 that each features three different dynamics. The expressions have been centered and reduced for this representation.

For the inference of the random effects, Best Linear Unbiased Predictor (BLUP) are used [52], giving access to estimations of a single profile for each gene among a gene set, in each patient. As a result, the estimations from the mixed model are shrunken towards the average expression inside the gene set. This shrinkage occurs when the residuals variability is relatively large compared to the the random effects estimated variances [52]. The mixed model uses the repeated pattern of the longitudinal measurements to structure the variation. Its estimations give smoother trajectories for the genes than the raw data, which makes the general evolution of the set clearer [53], as it can be seen in Figs 3 and 4.

### Characterization and visualization of dynamics

#### Dynamic of a significant gene set

Once a gene set *S* has been identified as significant through the previous mixed likelihood ratio statistics, a summary of its dynamic over time is needed. However, due to the possible heterogeneity of *S*, giving a summary representation of *S* dynamic is not obvious. We propose to automatically identify the number of trends in a significant gene set from the fit of the model. Predicted gene expressions from the linear mixed model are clustered, and the optimal number of trends is selected with the gap statistic [54]. It is a formalization of the elbow criterion for the within-cluster variance. In order to determine the optimal partition of each gene set here, the gap statistics is applied onto a hierarchical clustering of gene expressions inside each gene set. Then the median within each of the identified clusters can summarize each trend. Therefore, gene sets are actually split when heterogeneous, before being summarized. The predicted gene expression from the linear mixed model is used for this (and not the observed expression) because smoothness of trajectories facilitates classification [53]. Examples of such representations are given in Figs 3 and 4.

#### Global dynamics

**Fig 5:**
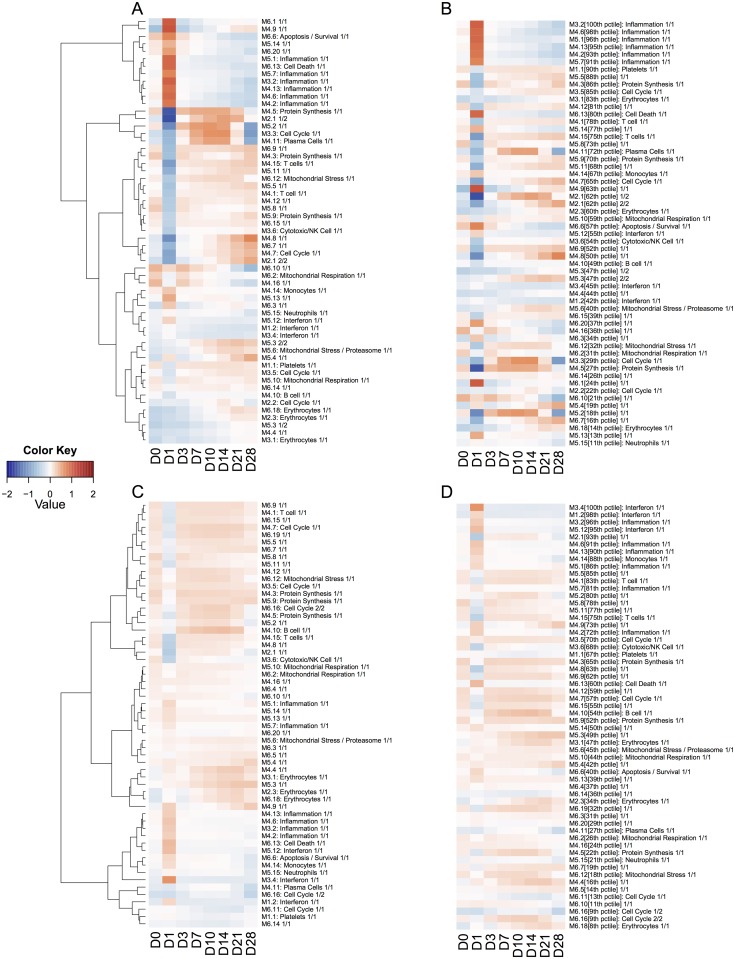
Heatmap of estimated dynamics from the significant gene sets among the 62 investigated gene sets when comparing vaccines arms to placebo arm. The median estimated gene expression over the patients is used for each trend. Each trend has seen its values reduced (so that its variance is 1) in order to make the dynamics more comparable. Each row is a group of gene having the same trend inside a gene set, and each column is a time point. The color key represents the median of the standardized estimation of gene expression over the patients for a given trend in a significant gene set. It becomes red as median expression is up-regulated or blue as it is down-regulated compared to the value in the placebo (saline) at the same time. A and C show the hierarchically clustered trends for pneumoccocal and flu respectively. B and D show the same trends but instead ranked by decreasing likelihood ratio percentiles of the associated gene set, for pneumoccocal and flu respectively.

Most often, TcGSA will be used to investigate a large number of gene sets (from a few hundreds to a few thousands). This multiplicity can make visualization of the results more challenging, in addition of requiring a multiple testing correction. TcGSA is designed to identify gene sets that shows a simultaneous evolution of gene expression, but possibly of a small intensity. The method can therefore be quite sensitive, and it can be of interest to rank the significant gene sets to identify the most acute signals. The likelihood ratio provides insight on the magnitude of the variation of each gene set. The percentile of their corresponding likelihood ratio gives an idea of the importance of the variation for a significant gene set. Examples of such representations are given in Fig 5.

### Implementation

The TcGSA method has been implemented in R as a package *TcGSA*, whose latest release is available from the CRAN repository (http://cran.r-project.org/web/packages/TcGSA/index.html).

## Results

### Motivating example: The DALIA-1 trial

The DALIA-1 trial is a phase 1 therapeutic HIV vaccine trial whose details are described on http://clinicaltrials.gov (ClinicalTrial.gov identifier: NCT00796770) and in [5]. The vaccine candidate was based on ex-vivo generated interferon-*α* dendritic cells loaded with HIV-1 lipopeptides and activated with lipopolysaccharide. The objectives of the trial were to evaluate the safety of the strategy and to evaluate the immune response to the vaccine. For the purpose of the present paper, we focus on the gene expression component of this study. Gene abundance in whole blood was measured through Illumina HumanHT-12 v4 Expression BeadChips.

#### The DALIA-1 trial design

**Fig 6:**
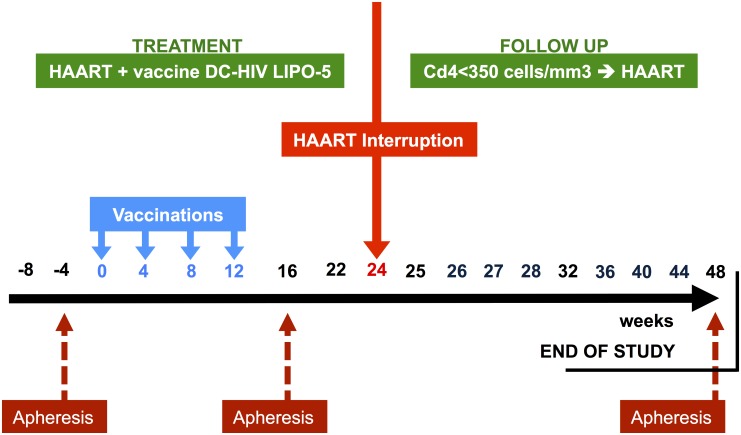
DALIA-1 trial design. Gene expression was measured at each time point, represented by a week number above the time axis. The trial was composed of two separated stages: (1) the treatment phase, during which the patients were vaccinated but remained under antiretroviral treatment; and(2) the follow- up phase commencing after the week 24 antiretroviral treatment interruption. Those two phases will be referred as pre-ATI and post-ATI respectively. The three apheresis time points were removed from the analysis due to a possible effect of the apheresis on the gene expression samples, and so was the first measurement (week -8) occurring right at the inclusion in the study.

All of the nineteen HIV infected patients received the therapeutic vaccine while under antiretroviral treatment. The patients received four injections at week 0, 4, 8 and 12. This vaccination period was followed by an antiretroviral treatment interruption (ATI) at week 24. The patients were followed up to week 48. Antiretroviral treatment was resumed from week 24 to week 48 at any time under the following criteria: i) if the patients or their doctors wished so; ii) if CD4+ T-cell count was < 350 cells/*μ*L and < 25% of total lymphocytes. Fourteen time points (five in pre-ATI from week 0 to week 22, and nine in post-ATI from week 24 to week 44) were used for this analysis (see Fig 6). One patient was removed from the analysis as his/her antiretroviral treatment compliance was irregular during the vaccination phase.

In the following analysis, two distinct datasets were considered: pre-ATI and post-ATI. The two datasets were normalized separately—via a normal-exponential convolution model [55, 56], followed by the application of the *ComBat* method [57] to correct for batch effects. Splitting the data allows us to study separately the vaccine effect and the treatment interruption, otherwise the ATI effect would mask any noticeable vaccine effect, because of the huge modification of gene expression related to viral replication [1, 2]. We investigated the gene sets defined by Chaussabel et al. [13], which are oriented towards the immune system. The definition and annotations of those 260 gene sets (called ‘Modules’) are available online (http://www.biir.net/public_wikis/module_annotation/V2_Trial_8_Modules).

#### Pre-ATI: The vaccination phase

During the vaccination period, a standard gene-by-gene mixed model analysis, with a cubic polynomial function of time, did not found any significant change of gene abundance at a 5% False Discovery Rate (see Table 1). However, during this vaccination phase, cytokines production analysis of the same blood samples (as measured by Luminex or intracellular staining) have showed that a response was induced by the vaccine at week 16 [5]. Therefore, one expected to observe a signal at the gene expression level between week 0 and week 16, the gene expression preceding molecular activation. Although the measurements were not performed in the hours or days following vaccination, the changes of gene abundance may reflect a change of the equilibrium of the overall expression in some gene sets. This kind of results has already been reported in cross-sectional studies [58]. Likewise, GSEA for time-series did not identify any significant gene set during vaccination. This can be explained by the lack of power of GSEA for time-series, as this method does not take into account the repeated structure of the data and is not suitable for longitudinal measurements. Finally, CAMERA did not identified any significant gene set either, in spite of testing a competitive null hypothesis.

**Table 1:** Number of significant units in DALIA-1 at a FDR of 5%.

	Pre-ATI	Post-ATI	Units
Gene-by-gene	0	3,389	probes ^a^
GSEA for time series	0	67	genes set ^b^
CAMERA	0	2	genes set ^b^
TcGSA linear	23	203	gene sets ^b^
TcGSA cubic polynom.	69	216	gene sets ^b^
TcGSA splines	68	219	gene sets ^b^

^a^ 32,978 probes investigated after filtering

^b^ 260 immune-related gene sets investigated (29 gene sets were automatically discarded because less than 10 probes were observed)

We applied the Time Course Gene set Analysis (see Methods) that allows to detect any change over time of gene abundance inside a gene set by detecting either trends over time or heterogeneity between gene dynamics. Fitting the Eq (1) with a cubic polynomial function of time, 69 gene sets out of 260 turned out to vary significantly. Fig 3 displays the raw sample-normalized and batch-corrected) and estimated gene expressions of 3 of the significant gene sets identified by TcGSA: T-cell, inflammation and B-cell gene sets. The identification of gene sets such as M4.1: T-cell (that includes CD402, CCR7, BCl2) was expected with regards to the CD4 T-cell response observed at Week 16 [5]. Also, the gene sets M4.6: inflammation and M6.7: B-cell are good examples of how smoothing from the estimations can give a much clearer dynamic pattern compared to the raw expression (see Methods).

#### Post-ATI: After antiretroviral treatment interruption

Eq (1) was then fitted to the data after antiretroviral treatment interruption that occurred at week 24: 216 gene sets out of 260 were found to be significant with a cubic polynomial function of time. Fig 4 displays the raw and estimated expressions of nine of those significant gene sets. It features heterogeneous gene sets, such as M4.16 and M7.1, which are both also good examples of the shrinkage that occurs with the estimations (see Methods). Meanwhile, GSEA for time series identifies 67 significant, whereas CAMERA identified only 2. These results are consistent with the lower statistical power found in our simulation study.

The large number of significant gene sets post-ATI illustrates the tremendous impact of the treatment interruption on the organism. Followed by a viral rebound, the treatment interruption is indeed a major event that triggers the expression of thousand of genes. Indeed, a gene-by-gene analysis revealed 3,389 significant probes (more than 10% of all the investigated probes—an unusually high number of differentially expressed genes). The immune system is very much in demand during the viral rebound. Therefore most of the gene sets from the Modules defined by Chaussabel et al. [13] are activated, as they are tightly linked with the immune system activity. Of particular interest are the three gene sets M1.2, M3.4 and M5.12 which are all annotated as *interferon*-related. These three gene sets exhibit similar dynamics (see Fig 4). Such a timely upregulation was expected, as it is associated to the viral rebound after treatment interruption and was previously reported [1, 2]. The gene set M3.4 is also linked with *antiviral response*.

### Another application: Influenza and pneumococcal vaccines responses

In a recent paper, Obermoser et al. [6] investigated the response to influenza and pneumococcal vaccines in healthy individuals at the gene expression level.

#### Study design

Healthy, young adults were randomly split in three groups of six volunteers each, receiving either a 2009–2010 seasonal influenza vaccine (Fluzone), a 23-valent pneumococcal vaccine (Pneumovax23), or a placebo (saline injections). Blood samples were collected at days -7, 0, 1, 3, 7, 10, 14, 21, and 28 to measure gene expression in whole blood. A more detailed description of the study can be found in Obermoser et al. [6].

#### Original analysis

In their modular analysis, Obermoser et al. [6] focused on 62 of the 260 available gene sets defined in Chaussabel et al. [13]. They investigated the changes of gene expression in those 62 gene sets for each of the seven time points from day 1 to day 28 in regards of the baseline, that was considered as the average of the two measurements at days -7 and 0. So hierarchical structure of the data was not taken into account. The three arms (saline, flu and pneumococcal) were analyzed separately, and only significant gene sets at day 1 and day 7 (not further on) are presented in their paper. Changes in eight gene sets were common to both vaccines: M4.6 (inflammation), M6.6 and M6.13 (apoptosis/cell death) and modules M4.1 and M4.15 (T cells), M4.3 (protein synthesis), M5.11, and M6.9 (no functional annotation). Nine gene sets were uniquely changing after the influenza vaccine, three were associated with antiviral responses (M1.2, M3.4, M5.12) and included genes coding for interferon (IFN)-inducible molecules. Six gene sets were uniquely responsive to the pneumococcal vaccine. Of these, five were modules including genes associated with inflammation: M3.2, M4.2, M4.13, M5.1 and M5.7.

#### TcGSA results

To compare the gene expression at the gene set level between the vaccine arm (flu or pneumococcal) and the placebo (saline) arm, we applied TcGSA on these data using Eq (2) (for each vaccine separately). In both vaccines, a large response is observed at Day 1. To avoid smoothing down the expression at *t*_*i*_ = 1, we used the following function of time to model the dynamic evolution of gene expression:

$$\begin{array}{c} f_{m}\left(t_{i}\right)=\left(\eta_{m}+h_{g}\right){𝟙}_{\left\{t_{i}=1\right\}}+\left(\eta_{m}^{\prime }+h_{g}^{\prime }\right){𝟙}_{\left\{t_{i}\neq 1\right\}} \end{array}$$

with $\left(h_{g},h_{g}^{\prime })∼𝓝(0,\Sigma_{h}\right)$, and *m* the group (either vaccine or placebo).

Most of the 62 investigated gene sets presented a significantly different evolution in vaccine arms compared to the placebo arm. Globally, the intensity of the response was stronger with the pneumoccocal vaccine than with the flu vaccine (Fig 5). The early response induced by the pneumococcal vaccine was dominated by inflammation whereas the top signal triggered by the flu vaccine involved an interferon signature (Fig 5B and 5D). In both vaccine, a T-cell response was also visible. In the pneumoccocal vaccine, a plasma cell signal, in association with cell cycle gene sets (Figs 5A and 5C), started at Day 7 until Day 14. This plasma blast signal was much less clear in the flu vaccine (Figs 5B and 5D). This is in agreement with the results of Obermoser et al. modular analysis.

TcGSA offers an extended and appropriate hierarchical analysis of these data. It provides a truly longitudinal insight into the vaccine responses, that are intrinsically compared to the placebo response. One of the main difference from the results presented in Obermoser et al. paper [6] is that, according to our analysis, the inflammation gene sets (M3.2, M4.13, M5.1 and M5.7) were also involved with the flu vaccine and were not specific to the pneumoccocal vaccine. This result is important as it means that both vaccine involved these inflammatory pathways. This result was not obvious from the original analysis because their approach was less powerful compared to TcGSA.

### Assessment of statistical performances on simulated data

In order to assess the behavior of the proposed method, a simulation study of TcGSA has been performed. The simulation scheme was chosen to be very close to the motivating exemple: the DALIA-1 trial. In each simulation run, gene expression data was simulated for 20 patients at 8 time points. 5,000 genes were simulated, divided into 100 non overlapping gene sets of 50 genes each. Each of the 100 gene sets was either simulated under (H_0_) or (H_1_). The proportion of genes under (H_1_) varied between 0%, 27% (which corresponds to results found in pre-ATI) and 85% (which corresponds to results found in post-ATI). When there are gene sets simulated under (H_1_), 75% of those were homogeneous (simulated with parameters close to those estimated for gene set M1.2 in DALIA post-ATI—see Fig 4) while the remaining 25% were heterogeneous (simulated with parameters close to those estimated for gene set M7.1 in DALIA post-ATI—see Fig 4).

Statistical performances of the proposed method are presented in Table 2. Without correcting for the fact that 100 gene set were investigated by TcGSA at each simulation runs, the average Type I error (the probability of rejecting H_0_ given that H_0_ is actually true) over a hundred runs was between 0.03 and 0.07 depending on the situation. But as soon as a control of the FDR was used, the Type-I error rate dropped well below 1%, regardless of the flexibility of the time function estimated (linear or cubic polynomials). The average statistical power (the probability of rejecting H_0_ given that H_0_ is actually false) is very good, always above 0.8 (dropping a little bit after multiple testing correction as expected).

**Table 2:** Assessment of statistical performances through a simulation study.

Percentage of simulated gene sets under H_1_	Method	Type I error	Type I error after MTC*	Statistical power	Statistical power after MTC*
0%	TcGSA (linear)	0.0394	0.0002	-	-
0%	TcGSA (cubic)	0.0649	0.0004	-	-
0%	globalANCOVA (perm)	0.0483	0.0001	-	-
0%	globalANCOVA (approx)	0.0006	0	-	-
0%	GSEA for time series	0	0	-	-
0%	CAMERA	0	0	-	-
27%	TcGSA (linear)	-	-	0.883	0.829
27%	TcGSA (cubic)	-	-	0.882	0.810
27%	globalANCOVA (perm)	-	-	0.787	0.706
27%	globalANCOVA (approx)	-	-	0.660	0.510
27%	GSEA for time series	-	-	0.459	0.214
27%	CAMERA	-	-	0.374	0.109
85%	TcGSA (linear)	-	-	0.885	0.847
85%	TcGSA (cubic)	-	-	0.882	0.833
85%	globalANCOVA (perm)	-	-	0.785	0.728
85%	globalANCOVA (approx)	-	-	0.660	0.549
85%	GSEA for time series	-	-	0.289	0.074
85%	CAMERA	-	-	0.177	0.013

* Multiple Testing Correction: performed via Benjamini-Yekutieli procedure with a 5% threshold.

In each simulation, 100 gene sets are simulated and significance level *α* = 5% is applied. This table displays the Type I error and the statistical power means over a hundred simulation runs for 3 different situations (0%, 27% and 85% of simulated gene sets are simulated under *H*_1_). Whenever the percentage of gene sets simulated under *H*_1_ is not null, 25% of the gene sets simulated under H_1_ are heterogeneous, the remaining 75% being homogeneous. Type I error is the probability of rejecting H_0_ given that H_0_ is true, i.e. for declaring a gene set significant when it actually is not. Statistical power is the probability of rejecting H_0_ given that H_1_ is true, i.e. for declaring a gene set significant when it actually is. Four methods are evaluated: i) TcGSA, the proposed approach, fitted either with a linear or with a cubic function of time; ii) the GlobalANCOVA procedure [29] in which p-values are either computed by permutation (10,000) or approximated; iii) the GSEA for time series [14], iv) CAMERA [31]. Default values are used for the various methods (see S1 Text and S1 Software).

Three other methods were also evaluated on those simulations, namely globalANCOVA [29] (using either permutations or an approximation to compute p-values), GSEA for time series and CAMERA. Their statistical performances are also presented in Table 2. Type I error is always well controlled by those three methods. However, both CAMERA and GSEA for time series exhibit very low statistical power (as low as 10 times less than TcGSA after multiple testing correction when there is a high proportion of significant gene sets). globalANCOVA, whose global null hypothesis is not so different from the one tested in TcGSA, performs quite well in terms of statistical power. Nonetheless it is still about 10% below TcGSA performances.

Those simulation results confirm that the higher number of selected gene sets by TcGSA in the two real-life examples presented in this paper are mainly due to the increased power of gene set analysis over gene-by-gene analysis (when repeated structure of the measurement is properly accounted for), and not to a large number of false positives.

## Discussion

In this paper, we present a method to analyze repeated measurements of gene expression using a gene set approach. Provided that the definition of the gene sets is relevant, this method helps with detecting and interpreting subtle changes of gene expression over time. In our applications where the same definition of gene sets has been applied, we were able to compare the response to several vaccines (against HIV, Influenza and Pneumococcus). Interestingly, we found common pathways that were triggered by all three vaccines, mostly related to inflammation, as well as pathways specific to each vaccine.

The capacity of the proposed approach to detect subtle changes of gene expression is due to two main factors: i) the use of a predefined gene sets that are functionally related ii) the use of all available information, taking advantage of repeated measurements using mixed models. Measurements of gene expression data in longitudinal studies may be missing because of missed visits or poor quality of the samples, leading to unbalanced data. Missing at random (MAR) processes (i.e. when the probability of missing data is associated to the previously measured information) may lead to biased estimates when using least squares or generalized estimating equations [37]. TcGSA can cope with such issues because of the use of Maximum Likelihood to estimate the parameters of the mixed models. This is an advantage of the TcGSA approach over those of Hummel et al. [29] or Nueda et al. [32].

An increasing number of gene sets databases are available, such as KEGG [11], Gene Ontology [12], Modules [13]. An immune related subset of Gene Ontology as well as an immune related subset of KEGG pathway have been used in additional analyses (see S3 Fig). The choice of the database used for the analysis impacts the interpretability but also the limitations of TcGSA. The efficiency of TcGSA will vary according to the number of genes represented in each gene set. The size of a given gene set has an impact on its significance, as the more genes it includes, the more likely a significant variation will be detected. The average size of the Chaussabel’s V2 modules is 55 genes. 17% of the 260 modules include more than 100 genes, and 31% less than 20 genes. For small gene sets, the normality assumptions of random effects of the Eqs (1), (1bis) and (2) are questionable (even though inference should still be robust [59]). Nevertheless, even though we expect that the models could be misspecified in many cases (if not all), the objective of such an analysis is to detect any significant variation over time (in the spirit of Shahbaba et al. [19], a significant variability of the trajectories between the genes inside a gene set indicates a change over time regardless of the fixed effects specification). The use of flexible time functions may help to get a better fit of dynamics although beyond cubic polynomials it did not have a substantial impact in our motivating example—see Table 1. These results vary according to the dataset and the number of time points available, and we recommend to try several models to check the robustness of the results.

TcGSA focuses on self-contained hypothesis. While competitive gene sets tests are appropriate in the presence of a strong signal (e.g. when comparing pre-ATI versus post-ATI), self-contained approaches are able to detect more subtle and sparse signals (e.g in pre-ATI). The choice between a competitive or a self-contained approach is therefore highly dependent on the biological question.

Several extensions of the TcGSA are possible for its use in other contexts. One can also model time trends with a random effect grouped on the patient level as of *γ*_*p*,⋅_, instead of on the gene level as in Eqs (1), (1bis) and (2). This identifies gene sets whose dynamic differs across the patients. This option is also implemented in the *TcGSA* R package. TcGSA could easily be adapted to mRNA counts data. In that case, generalized linear mixed effects models could be used, with a Poisson distribution for instance, instead of linear mixed effect models that rely on a Gaussian assumption. Or else, precision weights estimated with voom [60] could also be used to model the mean-variance relationship while fitting the proposed linear mixed effect directly on log-counts data from RNA-seq, thus taking into account heteroscedasticity.

In conclusion, the method presented gives a solution for the full exploitation of any repeated measurements of gene expression data based on a gene set analysis where a great sensibility to detect subtle change, while controlling false discovery, is needed.

## Supporting Information

S1 Fig Density plot for both the 100,000 simulations under the null and a 100,000 sample of the corresponding *χ* ^2^ mixture approximation. (EPS)

S2 Fig Quantile-quantile plot comparing the 100,000 simulations under the null to a 100,000 sample of the corresponding *χ* ^2^ mixture approximation. (TIFF)

S3 Fig Comparison of TcGSA results on DALIA-1 trial data for the three gene sets databases considered. During pre-ATI, 3 out of 75 gene sets were significant in the subset of KEGG, and 0 out of 131 in the subset of GO. During post-ATI, 73 out of 75 gene sets were significant in the subset of KEGG, and 101 out of 131 in the subset of GO. 2 gene sets the subset of KEGG and 20 from the subset of GO were automatically discarded because less than 10 probes or more than 500 probes were observed.
(TIFF)

S1 Table Selected KEGG pathways for investigating DALIA-1. Hand picked KEGG pathways [11] of interest for investigating DALIA-1 trial data.
(PDF)

S2 Table Selected GO pathways for investigating DALIA-1. Hand picked Gene Ontology pathways [12] of interest for investigating DALIA-1 trial data.
(PDF)

S1 Text Supporting Information about TcGSA. (PDF)

S1 Software R files helping to reproduce the results from this article. (ZIP)

S1 File The raw p-value and the adjusted false discovery rate—calculated with the Benjamini-Yiekutieli procedure—in pre-ATI in the DALIA-1 trial. (TXT)

S2 File The raw p-value and the adjusted false discovery rate—calculated with the Benjamini-Yiekutieli procedure—in post-ATI in the DALIA-1 trial. (TXT)

S3 File The raw p-value and the adjusted false discovery rate—calculated with the Benjamini-Yiekutieli procedure—for the pneumoccocal vaccine. (TXT)

S4 File The raw p-value and the adjusted false discovery rate—calculated with the Benjamini-Yiekutieli procedure—for the flu vaccine. (TXT)
