# Correction: Time-Course Gene Set Analysis for Longitudinal Gene Expression Data

**DOI:** 10.1371/journal.pcbi.1004446

**Published:** 2015-08-21

**Authors:** The PLOS Computational Biology Staff

There is an error in the Affiliations section: Boris P. Hejblum and Rodolphe Thiébaut are not affiliated with #5, the Baylor Institute for Immunology Research, Dallas, Texas, United States of America.
