# Supplementary figures and images for: Time-Course Gene Set Analysis for Longitudinal Gene Expression Data
Source: PLoS Comput Biol. 2015 Jun 25;11(6):e1004310. doi: 10.1371/journal.pcbi.1004310 (PMC4482329; doi:10.1371/journal.pcbi.1004310)

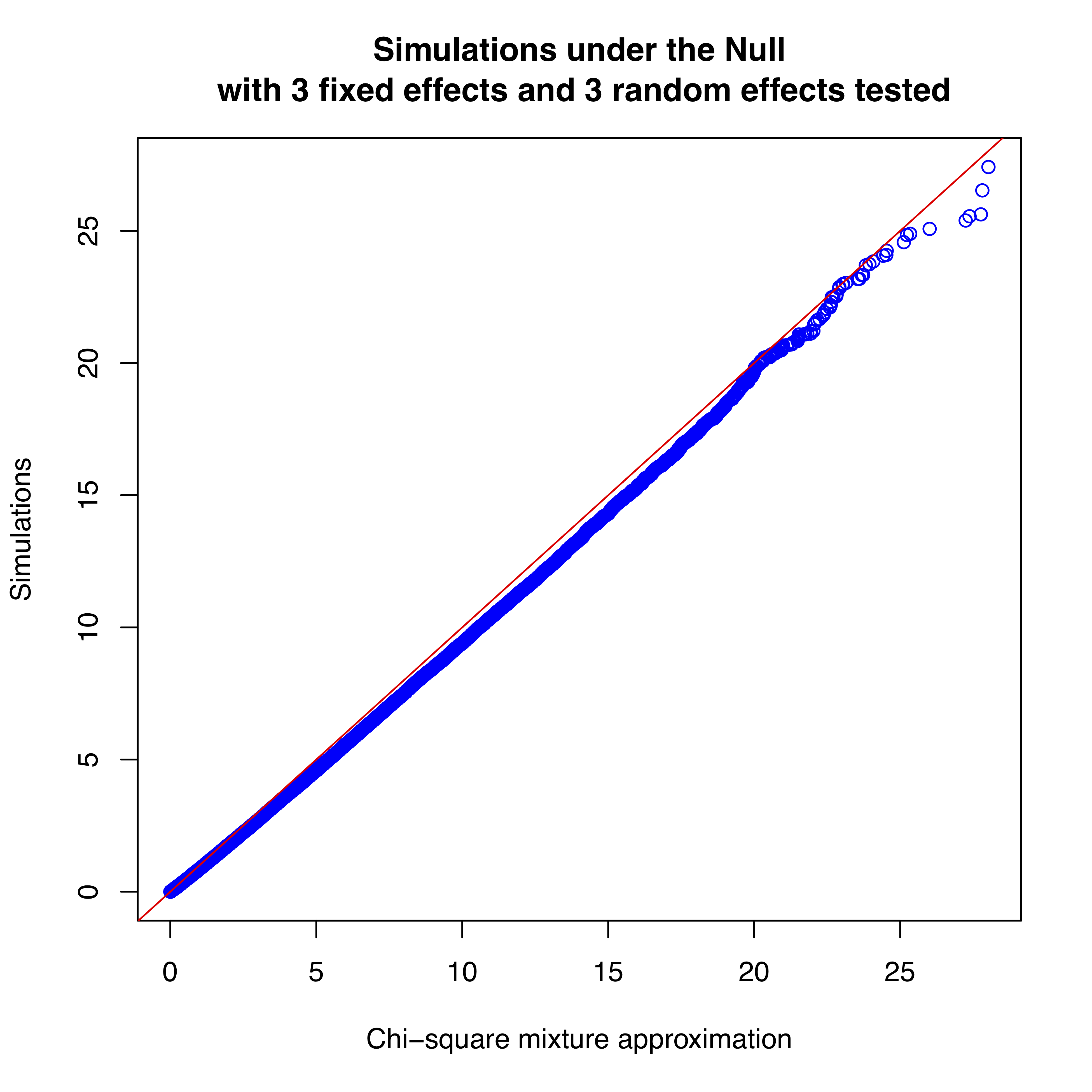

Supplement: S2 Fig — (TIFF) [file pcbi.1004310.s002.tiff]

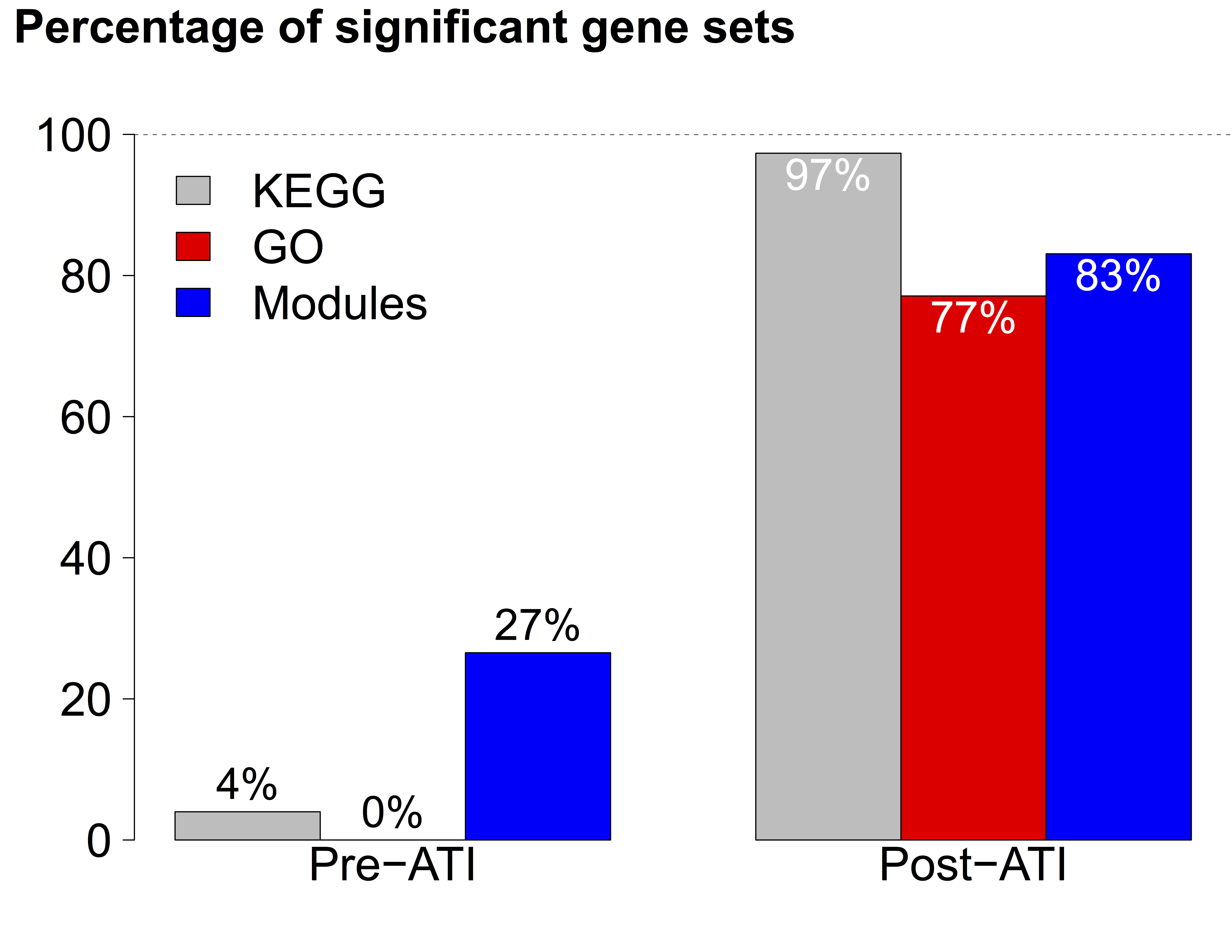

Supplement: S3 Fig — During pre-ATI, 3 out of 75 gene sets were significant in the subset of KEGG, and 0 out of 131 in the subset of GO. During post-ATI, 73 out of 75 gene sets were significant in the subset of KEGG, and 101 out of 131 in the subset of GO. 2 gene sets the subset of KEGG and 20 from the subset of GO were automatically discarded because less than 10 probes or more than 500 probes were observed. (TIFF) [file pcbi.1004310.s003.tiff]
