# Supplementary material for: Time-Course Gene Set Analysis for Longitudinal Gene Expression Data
Source: PLoS Comput Biol. 2015 Jun 25;11(6):e1004310. doi: 10.1371/journal.pcbi.1004310 (PMC4482329; doi:10.1371/journal.pcbi.1004310)
Supplement: S1 Table — Hand picked KEGG pathways [11] of interest for investigating DALIA-1 trial data. (PDF) [file pcbi.1004310.s004.pdf]

Table S1. Selected KEGG pathways for investigating DALIA-1

	KEGG ID	Description
1	path:hsa04910 †	Endocrine System:Insulin signaling pathway
2	path:hsa04920 †	Endocrine System:Adipocytokine signaling pathway
3	path:hsa03320 †	Endocrine System:PPAR signaling pathway
4	path:hsa04912 †	Endocrine System:GnRH signaling pathway
5	path:hsa04914 †	Endocrine System:Progesterone-mediated oocyte maturation
6	path:hsa04916 †	Endocrine System:Melanogenesis
7	path:hsa04614 †	Endocrine System:Renin-angiotensin system
8	path:hsa04640 †	Immune System:Hematopoietic cell lineage
9	path:hsa04610 †	Immune System:Complement and coagulation cascades
10	path:hsa04620 †	Immune System:Toll-like receptor signaling pathway
11	path:hsa04621 †	Immune System:NOD-like receptor signaling pathway
12	path:hsa04622 †	Immune System:RIG-I-like receptor signaling pathway
13	path:hsa04623 †	Immune System:Cytosolic DNA-sensing pathway
14	path:hsa04650 †	Immune System:Natural killer cell mediated cytotoxicity
15	path:hsa04612 †	Immune System:Antigen processing and presentation
16	path:hsa04660 †	Immune System:T cell receptor signaling pathway
17	path:hsa04662 †	Immune System:B cell receptor signaling pathway
18	path:hsa04664 †	Immune System:Fc epsilon RI signaling pathway
19	path:hsa04666 †	Immune System:Fc gamma R-mediated phagocytosis
20	path:hsa04670 †	Immune System:Leukocyte transendothelial migration
21	path:hsa04672 †	Immune System:Intestinal immune network for IgA production
22	path:hsa04062 †	Immune System:Chemokine signaling pathway
23	path:hsa04510 †	Cell Communication:Focal adhesion
24	path:hsa04520 †	Cell Communication:Adherens junction
25	path:hsa04530 †	Cell Communication:Tight junction
26	path:hsa04540 †	Cell Communication:Gap junction
27	path:hsa04110 †	Cell Growth and Death:Cell cycle
28	path:hsa04114 †	Cell Growth and Death:Oocyte meiosis
29	path:hsa04210 †	Cell Growth and Death:Apoptosis
30	path:hsa04115 †	Cell Growth and Death:p53 signaling pathway
31	path:hsa04144 †	Transport and Catabolism:Endocytosis
32	path:hsa04145 †	Transport and Catabolism:Phagosome
33	path:hsa04142*†	Transport and Catabolism:Lysosome
34	path:hsa04146 †	Transport and Catabolism:Peroxisome
35	path:hsa04140 †	Transport and Catabolism:Regulation of autophagy
36	path:hsa04810 †	Cell Motility:Regulation of actin cytoskeleton
37	path:hsa02010 †	Membrane Transport:ABC transporters
38	path:hsa04010 †	Signal Transduction:MAPK signaling pathway
39	path:hsa04012 †	Signal Transduction:ErbB signaling pathway
40	path:hsa04310 †	Signal Transduction:Wnt signaling pathway
41	path:hsa04330 †	Signal Transduction:Notch signaling pathway
42	path:hsa04340 †	Signal Transduction:Hedgehog signaling pathway
43	path:hsa04350 †	Signal Transduction:TGF-beta signaling pathway
44	path:hsa04370 †	Signal Transduction:VEGF signaling pathway
45	path:hsa04630 †	Signal Transduction:Jak-STAT signaling pathway
46	path:hsa04064 †	Signal Transduction:NF-kappa B signaling pathway
47	path:hsa04020 †	Signal Transduction:Calcium signaling pathway
48	path:hsa04070 †	Signal Transduction:Phosphatidylinositol signaling system
49	path:hsa04151 †	Signal Transduction:PI3K-Akt signaling pathway
50	path:hsa04150 †	Signal Transduction:mTOR signaling pathway
51	path:hsa04080 †	Signaling Molecules and Interaction:Neuroactive ligand-receptor interaction
52	path:hsa04060 †	Signaling Molecules and Interaction:Cytokine-cytokine receptor interaction
53	path:hsa04512 †	Signaling Molecules and Interaction:ECM-receptor interaction
54	path:hsa03030 †	Replication and Repair:DNA replication
55	path:hsa03410 †	Replication and Repair:Base excision repair
56	path:hsa03420 †	Replication and Repair:Nucleotide excision repair
57	path:hsa03430 †	Replication and Repair:Mismatch repair
58	path:hsa03440 †	Replication and Repair:Homologous recombination
59	path:hsa03450	Replication and Repair:Non-homologous end-joining
60	path:hsa03460 †	Replication and Repair:Fanconi anemia pathway
61	path:hsa03060 †	Folding, Sorting and Degradation:Protein export
62	path:hsa04141 †	Folding, Sorting and Degradation:Protein processing in endoplasmic reticulum
63	path:hsa04130 †	Folding, Sorting and Degradation:SNARE interactions in vesicular transport
64	path:hsa04120 †	Folding, Sorting and Degradation:Ubiquitin mediated proteolysis
65	path:hsa04122	Folding, Sorting and Degradation:Sulfur relay system
66	path:hsa03050 †	Folding, Sorting and Degradation:Proteasome
67	path:hsa03018 †	Folding, Sorting and Degradation:RNA degradation
68	path:hsa03010*†	Translation:Ribosome
69	path:hsa00970 †	Translation:Aminoacyl-tRNA biosynthesis
70	path:hsa03013 †	Translation:RNA transport
71	path:hsa03015 †	Translation:mRNA surveillance pathway
72	path:hsa03008 †	Translation:Ribosome biogenesis in eukaryotes
73	path:hsa03020 †	Transcription:RNA polymerase
74	path:hsa03022 †	Transcription:Basal transcription factors
75	path:hsa03040*†	Transcription:Spliceosome

*: significant (FDR<0.05) in pre-ATI   †: significant (FDR<0.05) in post-ATI
