# Supplementary material for: Time-Course Gene Set Analysis for Longitudinal Gene Expression Data
Source: PLoS Comput Biol. 2015 Jun 25;11(6):e1004310. doi: 10.1371/journal.pcbi.1004310 (PMC4482329; doi:10.1371/journal.pcbi.1004310)
Supplement: S2 Table — Hand picked Gene Ontology pathways [12] of interest for investigating DALIA-1 trial data. (PDF) [file pcbi.1004310.s005.pdf]

Table S2. Selected GO pathways for investigating DALIA-1

	GO ID		Description
1	GO:0002218	†	activation of innate immune response
2	GO:0006956	†	complement activation
3	GO:0002429	†	immune response-activating cell surface receptor signaling pathway
4	GO:0002758	†	innate immune response-activating signal transduction
5	GO:0019883	†	antigen processing and presentation of endogenous antigen
6	GO:0019884	†	antigen processing and presentation of exogenous antigen
7	GO:0048002	†	antigen processing and presentation of peptide antigen
8	GO:0002504	†	antigen processing and presentation of peptide or polysaccharide antigen via MHC class II
9	GO:0002475		antigen processing and presentation via MHC class Ib
10	GO:0002468		dendritic cell antigen processing and presentation
11	GO:0002578		negative regulation of antigen processing and presentation
12	GO:0002579	†	positive regulation of antigen processing and presentation
13	GO:0002577	†	regulation of antigen processing and presentation
14	GO:0002457		T cell antigen processing and presentation
15	GO:0002339		B cell selection
16	GO:0002263	†	cell activation involved in immune response
17	GO:0051607	†	defense response to virus
18	GO:0002432		granuloma formation
19	GO:0002434		immune complex clearance
20	GO:0043299	†	leukocyte degranulation
21	GO:0001909	†	leukocyte mediated cytotoxicity
22	GO:0019724	†	B cell mediated immunity
23	GO:0002228	†	natural killer cell mediated immunity
24	GO:0002707	†	negative regulation of lymphocyte mediated immunity
25	GO:0002708	†	positive regulation of lymphocyte mediated immunity
26	GO:0002706	†	regulation of lymphocyte mediated immunity
27	GO:0002456	†	T cell mediated immunity
28	GO:0002444	†	myeloid leukocyte mediated immunity
29	GO:0002704	†	negative regulation of leukocyte mediated immunity
30	GO:0002705	†	positive regulation of leukocyte mediated immunity
31	GO:0002703	†	regulation of leukocyte mediated immunity
32	GO:0002522		leukocyte migration involved in immune response
33	GO:0002698	†	negative regulation of immune effector process
34	GO:0008228		opsonization
35	GO:0002699	†	positive regulation of immune effector process
36	GO:0002697	†	regulation of immune effector process
37	GO:0002679	†	respiratory burst involved in defense response
38	GO:0002250	†	adaptive immune response
39	GO:0002367	†	cytokine production involved in immune response
40	GO:0006959	†	humoral immune response
41	GO:0002418	†	immune response to tumor cell
42	GO:0002437	†	inflammatory response to antigenic stimulus
43	GO:0006957	†	complement activation, alternative pathway
44	GO:0001867		complement activation, lectin pathway
45	GO:0002227		innate immune response in mucosa
46	GO:0045824	†	negative regulation of innate immune response
47	GO:0045089	†	positive regulation of innate immune response
48	GO:0045088	†	regulation of innate immune response
49	GO:0034341	†	response to interferon-gamma
50	GO:0034340	†	response to type I interferon
51	GO:0050777	†	negative regulation of immune response
52	GO:0002251	†	organ or tissue specific immune response
53	GO:0052555		positive regulation by organism of immune response of other organism involved in symbiotic interaction
54	GO:0002821	†	positive regulation of adaptive immune response
55	GO:0002922		positive regulation of humoral immune response
56	GO:0002839	†	positive regulation of immune response to tumor cell
57	GO:0002863	†	positive regulation of inflammatory response to antigenic stimulus
58	GO:0002830		positive regulation of type 2 immune response
59	GO:0002765	†	immune response-inhibiting signal transduction
60	GO:0002768	†	immune response-regulating cell surface receptor signaling pathway
61	GO:0052552		modulation by organism of immune response of other organism involved in symbiotic interaction
62	GO:0002819	†	regulation of adaptive immune response
63	GO:0002718	†	regulation of cytokine production involved in immune response
64	GO:0043309		regulation of eosinophil degranulation
65	GO:0002920	†	regulation of humoral immune response
66	GO:0002837	†	regulation of immune response to tumor cell
67	GO:0002861	†	regulation of inflammatory response to antigenic stimulus
68	GO:0033006	†	regulation of mast cell activation involved in immune response
69	GO:0043380		regulation of memory T cell differentiation
70	GO:0043313		regulation of neutrophil degranulation
71	GO:0045622	†	regulation of T-helper cell differentiation
72	GO:0002828	†	regulation of type 2 immune response
73	GO:0042092	†	type 2 immune response
74	GO:0002520		immune system development
75	GO:0002366	†	leukocyte activation involved in immune response

76	GO:0050902		leukocyte adhesive activation
77	GO:0042113	†	B cell activation
78	GO:0001767		establishment of lymphocyte polarity
79	GO:0001771		immunological synapse formation
80	GO:0002285	†	lymphocyte activation involved in immune response
81	GO:0030098	†	lymphocyte differentiation
82	GO:0046651	†	lymphocyte proliferation
83	GO:0030101	†	natural killer cell activation
84	GO:0051250	†	negative regulation of lymphocyte activation
85	GO:0031294	†	lymphocyte costimulation
86	GO:0050871	†	positive regulation of B cell activation
87	GO:0045621	†	positive regulation of lymphocyte differentiation
88	GO:0050671	†	positive regulation of lymphocyte proliferation
89	GO:0032816	†	positive regulation of natural killer cell activation
90	GO:0050870	†	positive regulation of T cell activation
91	GO:0050864	†	regulation of B cell activation
92	GO:0045619	†	regulation of lymphocyte differentiation
93	GO:0050670	†	regulation of lymphocyte proliferation
94	GO:0032814	†	regulation of natural killer cell activation
95	GO:0050863	†	regulation of T cell activation
96	GO:0050868	†	negative regulation of T cell activation
97	GO:0046634	†	regulation of alpha-beta T cell activation
98	GO:0046643	†	regulation of gamma-delta T cell activation
99	GO:2001188		regulation of T cell activation via T cell receptor contact with antigen bound to MHC molecule on antigen presenting cell
100	GO:0045580	†	regulation of T cell differentiation
101	GO:0042129	†	regulation of T cell proliferation
102	GO:0046631	†	alpha-beta T cell activation
103	GO:0001768		establishment of T cell polarity
104	GO:0046629	†	gamma-delta T cell activation
105	GO:0035709		memory T cell activation
106	GO:0002286	†	T cell activation involved in immune response
107	GO:0030217	†	T cell differentiation
108	GO:0042098	†	T cell proliferation
109	GO:0002274	†	myeloid leukocyte activation
110	GO:0002695	†	negative regulation of leukocyte activation
111	GO:0002696	†	positive regulation of leukocyte activation
112	GO:0043030	†	regulation of macrophage activation
113	GO:0033003	†	regulation of mast cell activation
114	GO:0030885		regulation of myeloid dendritic cell activation
115	GO:0001776	†	leukocyte homeostasis
116	GO:0050900	†	leukocyte migration
117	GO:0002262	†	myeloid cell homeostasis
118	GO:0002683	†	negative regulation of immune system process
119	GO:0050857	†	positive regulation of antigen receptor-mediated signaling pathway
120	GO:0060369		positive regulation of Fc receptor mediated stimulatory signaling pathway
121	GO:0002253	†	activation of immune response
122	GO:0002687	†	positive regulation of leukocyte migration
123	GO:0070426		positive regulation of nucleotide-binding oligomerization domain containing signaling pathway
124	GO:2000525		positive regulation of T cell costimulation
125	GO:0002645	†	positive regulation of tolerance induction
126	GO:0034123	†	positive regulation of toll-like receptor signaling pathway
127	GO:0002440	†	production of molecular mediator of immune response
128	GO:0002682		regulation of immune system process
129	GO:0002200	†	somatic diversification of immune receptors
130	GO:0045058	†	T cell selection
131	GO:0002507	†	tolerance induction

*: significant (FDR<0.05) in pre-ATI   †: significant (FDR<0.05) in post-ATI
