# Supplementary material for: Time-Course Gene Set Analysis for Longitudinal Gene Expression Data
Source: PLoS Comput Biol. 2015 Jun 25;11(6):e1004310. doi: 10.1371/journal.pcbi.1004310 (PMC4482329; doi:10.1371/journal.pcbi.1004310)
Supplement: S1 Text — (PDF) [file pcbi.1004310.s006.pdf]

Time-course Gene Set Analysis for Longitudinal Gene Expression Data – Supplementary Information

Boris P. Hejblum[1,2,3], Jason Skinner[4], Rodolphe Thiébaut[1,2,3,*]
1Univ. Bordeaux, ISPED, Centre INSERM U897-Epidemiologie-Biostatistique, F-33000 Bordeaux, France
2 INSERM, ISPED, Centre INSERM U897-Epidemiologie-Biostatistique, F-33000 Bordeaux, France
3 INRIA, Team SISTM, F-33000 Bordeaux, France
4 Baylor Institute for Immunology Research, Dallas, TX, USA
∗ E-mail: rodolphe.thiebaut@isped.u-bordeaux2.fr

List of Figures

S1 **Density plot for both the 100,000 simulations under the null and a 100,000 sample of the corresponding χ^2 mixture approximation.** 2
S2 **Quantile-Quantile plot comparing the 100,000 simulations under the null to a 100,000 sample of the corresponding χ^2 mixture approximation.** 2
S3 **Comparison of TcGSA results on DALIA-1 for the three gene sets databases considered.** During pre-ATI (vaccination phase of the DALIA-1 trial), 3 out of 75 gene sets were significant in the subset of KEGG, and 0 out of 131 in the subset of GO. During post-ATI, 73 out of 75 gene sets were significant in the subset of KEGG, and 101 out of 131 in the subset of GO. 2 gene sets the subset of KEGG and 20 from the subset of GO were automatically discarded because less than 10 probes or more than 500 probes were observed. 3

List of Tables

S1 Selected KEGG pathways for investigating DALIA-1 5
S2 Selected GO pathways for investigating DALIA-1 6

1 Approximation of the asymptotic distribution of the mixed likelihood ratio test under H_0

The χ^2 mixture distribution derived from the work of Self & Liang [3] is only an approximation of the distribution of the mixed Likelihood Ratio Test (mLRT) under the null hypothesis $H0$. We compared 100,000 LRTs computed on simulated gene sets (with similar settings as those of the DALIA-1 trial) under the null hypothesis (no effect of time, either as a fixed effect or a random effect, using a cubic polynomial function of time). As shown in Figures S1 and S2 , the approximation seems quite valid, even though the random effects (three functions of time) are not independent [4–6].

2 Assessment of the statistical properties of GSEA for time series

Following is the java call used for computing GSEA for time series results on the 100 simulated gene sets:

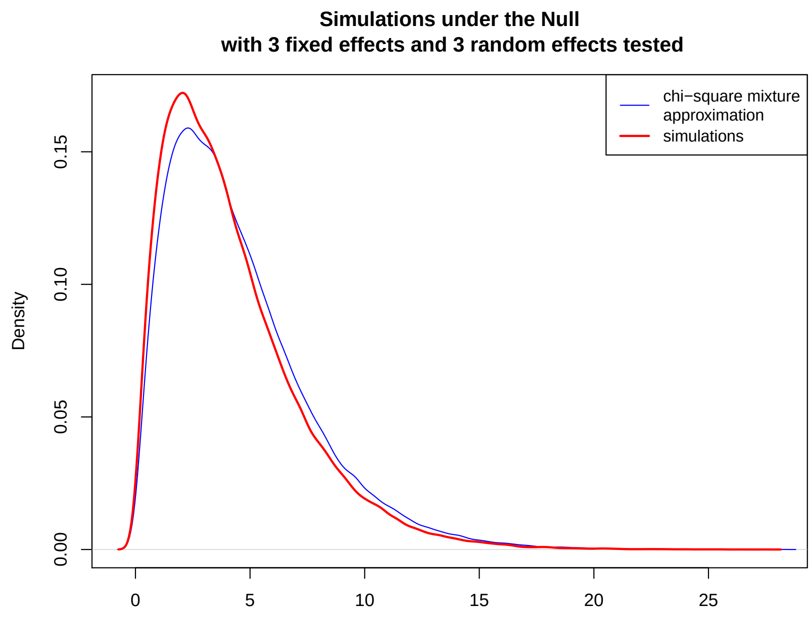

Figure S1. Density plot for both the 100,000 simulations under the null and a 100,000 sample of the corresponding χ^2 mixture approximation.

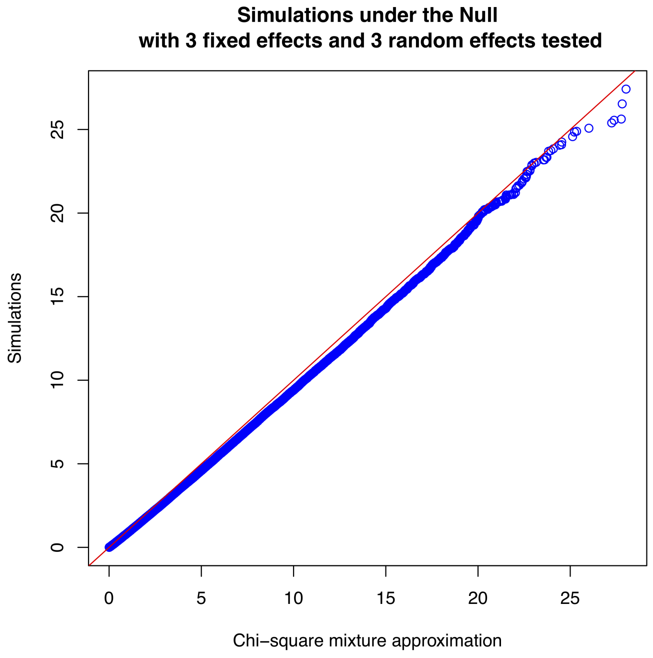

Figure S2. Quantile-Quantile plot comparing the 100,000 simulations under the null to a 100,000 sample of the corresponding χ^2 mixture approximation.

- ```
  java -cp gsea2-2.0.14.jar xtools.gsea.Gsea
  -res sim_expr.gct
  -cls sim_pheno.cls#timepoint
  -gmx sim_gmt.gmt
  -collapse false
  -metric Pearson
  ```

3 DALIA-1 TcGSA analysis compared for the whole blood modules, KEGG and Gene Ontology

In addition to utilizing the whole blood Illumina V2 modules [7] the DALIA-1 data were analyzed with two other databases: i) a subset of the KEGG [1] pathway database (see table S1) and ii) a subset of the GO [2] database (see table S2). Since the whole blood modules were derived from multiple independent datasets that encompass a wide range of immune related diseases (see `http://www.interactivefigures.com/dm3/vaccine-paper/faq.gsp` for more information), the 260 module gene sets are highly enriched in immune related annotations. This facilitates the interpretation of TcGSA findings in terms of describing immune changes in the blood associated with vaccination and viral rebound following treatment interruption. This explains why the modules are more sensitive in pre-ATI during the vaccination phase of the DALIA trial (see Figure S3). However, the viral rebound is such a cataclysm for the immune system that regardless of the database used, a large part of the gene sets are activated.

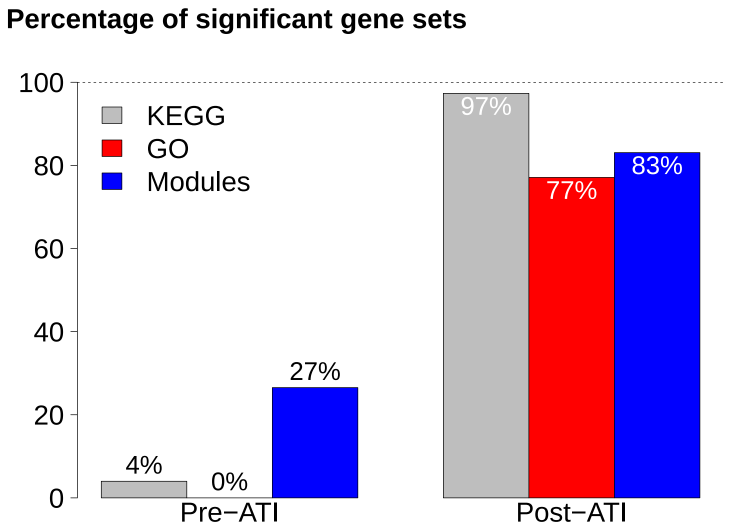

Figure S3. Comparison of TcGSA results on DALIA-1 for the three gene sets databases considered. During pre-ATI (vaccination phase of the DALIA-1 trial), 3 out of 75 gene sets were significant in the subset of KEGG, and 0 out of 131 in the subset of GO. During post-ATI, 73 out of 75 gene sets were significant in the subset of KEGG, and 101 out of 131 in the subset of GO. 2 gene sets the subset of KEGG and 20 from the subset of GO were automatically discarded because less than 10 probes or more than 500 probes were observed.
  ```

4 Software

The zip folder ReproducibleRFiles.zip contains R files helping to reproduce the results from the article.

5 Supplementary data

Four text files are provided along these supplementary information. They contain output results from TcGSA with Chaussabel's gene sets [7] on both example presented in the article:

- TcGSA_SupMat_preATI_LRTfdrSignificantGS.txt:
  the raw p-value and the ajusted false discovery rate – calculated with the Benjamini-Yiekutieli procedure [8] – in pre-ATI.
- TcGSA_SupMat_postATI_LRTfdrSignificantGS.txt:
  the raw p-value and the ajusted false discovery rate – calculated with the Benjamini-Yiekutieli procedure in post-ATI.
- TcGSA_SupMat_Pneumo_LRTfdrSignificantGS.txt:
  the raw p-value and the ajusted false discovery rate – calculated with the Benjamini-Yiekutieli procedure with the pneumoccocal vaccine.
- TcGSA_SupMat_Flu_LRTfdrSignificantGS.txt:
  the raw p-value and the ajusted false discovery rate – calculated with the Benjamini-Yiekutieli procedure with the flu vaccine.

References

1. Kanehisa M, Goto S (2000) KEGG: kyoto encyclopedia of genes and genomes. Nucleic acids research 28: 27–30.
2. Ashburner M, Ball CA, Blake JA, Botstein D, Butler H, et al. (2000) Gene ontology: tool for the unification of biology. The Gene Ontology Consortium. Nature genetics 25: 25–9.
3. Self SG, Liang Ky (1987) Asymptotic properties of maximum likelihood estimators and likelihood ratio tests under nonstandard conditions. Journal of the American Statistical Association 82: 605–610.
4. Stram DO, Lee JW (1994) Variance components testing in the longitudinal mixed effects model. Biometrics 50: 1171–1177.
5. Stram DO, Lee JW (1995) Corrections to "Variance components testing in the longitudinal mixed effects model" by D. O. Stram and J. W. Lee; 50, 1171-1177, 1994. Biometrics 51: 1196.
6. Molenberghs G, Verbeke G (2007) Likelihood Ratio, Score, and Wald Tests in a Constrained Parameter Space. The American Statistician 61: 22–27.
7. Chaussabel D, Quinn C, Shen J, Patel P, Glaser C, et al. (2008) A modular analysis framework for blood genomics studies: application to systemic lupus erythematosus. Immunity 29: 150–164.
8. Yekutieli D, Benjamini Y (2001) The control of the false discovery rate in multiple testing under dependency. The Annals of Statistics 29: 1165–1188.

Table S1. Selected KEGG pathways for investigating DALIA-1

	KEGG ID	Description
1	path:hsa04910 †	Endocrine System:Insulin signaling pathway
2	path:hsa04920 †	Endocrine System:Adipocytokine signaling pathway
3	path:hsa03320 †	Endocrine System:PPAR signaling pathway
4	path:hsa04912 †	Endocrine System:GnRH signaling pathway
5	path:hsa04914 †	Endocrine System:Progesterone-mediated oocyte maturation
6	path:hsa04916 †	Endocrine System:Melanogenesis
7	path:hsa04614 †	Endocrine System:Renin-angiotensin system
8	path:hsa04640 †	Immune System:Hematopoietic cell lineage
9	path:hsa04610 †	Immune System:Complement and coagulation cascades
10	path:hsa04620 †	Immune System:Toll-like receptor signaling pathway
11	path:hsa04621 †	Immune System:NOD-like receptor signaling pathway
12	path:hsa04622 †	Immune System:RIG-I-like receptor signaling pathway
13	path:hsa04623 †	Immune System:Cytosolic DNA-sensing pathway
14	path:hsa04650 †	Immune System:Natural killer cell mediated cytotoxicity
15	path:hsa04612 †	Immune System:Antigen processing and presentation
16	path:hsa04660 †	Immune System:T cell receptor signaling pathway
17	path:hsa04662 †	Immune System:B cell receptor signaling pathway
18	path:hsa04664 †	Immune System:Fc epsilon RI signaling pathway
19	path:hsa04666 †	Immune System:Fc gamma R-mediated phagocytosis
20	path:hsa04670 †	Immune System:Leukocyte transendothelial migration
21	path:hsa04672 †	Immune System:Intestinal immune network for IgA production
22	path:hsa04062 †	Immune System:Chemokine signaling pathway
23	path:hsa04510 †	Cell Communication:Focal adhesion
24	path:hsa04520 †	Cell Communication:Adherens junction
25	path:hsa04530 †	Cell Communication:Tight junction
26	path:hsa04540 †	Cell Communication:Gap junction
27	path:hsa04110 †	Cell Growth and Death:Cell cycle
28	path:hsa04114 †	Cell Growth and Death:Oocyte meiosis
29	path:hsa04210 †	Cell Growth and Death:Apoptosis
30	path:hsa04115 †	Cell Growth and Death:p53 signaling pathway
31	path:hsa04144 †	Transport and Catabolism:Endocytosis
32	path:hsa04145 †	Transport and Catabolism:Phagosome
33	path:hsa04142*†	Transport and Catabolism:Lysosome
34	path:hsa04146 †	Transport and Catabolism:Peroxisome
35	path:hsa04140 †	Transport and Catabolism:Regulation of autophagy
36	path:hsa04810 †	Cell Motility:Regulation of actin cytoskeleton
37	path:hsa02010 †	Membrane Transport:ABC transporters
38	path:hsa04010 †	Signal Transduction:MAPK signaling pathway
39	path:hsa04012 †	Signal Transduction:ErbB signaling pathway
40	path:hsa04310 †	Signal Transduction:Wnt signaling pathway
41	path:hsa04330 †	Signal Transduction:Notch signaling pathway
42	path:hsa04340 †	Signal Transduction:Hedgehog signaling pathway
43	path:hsa04350 †	Signal Transduction:TGF-beta signaling pathway
44	path:hsa04370 †	Signal Transduction:VEGF signaling pathway
45	path:hsa04630 †	Signal Transduction:Jak-STAT signaling pathway
46	path:hsa04064 †	Signal Transduction:NF-kappa B signaling pathway
47	path:hsa04020 †	Signal Transduction:Calcium signaling pathway
48	path:hsa04070 †	Signal Transduction:Phosphatidylinositol signaling system
49	path:hsa04151 †	Signal Transduction:PI3K-Akt signaling pathway
50	path:hsa04150 †	Signal Transduction:mTOR signaling pathway
51	path:hsa04080 †	Signaling Molecules and Interaction:Neuroactive ligand-receptor interaction
52	path:hsa04060 †	Signaling Molecules and Interaction:Cytokine-cytokine receptor interaction
53	path:hsa04512 †	Signaling Molecules and Interaction:ECM-receptor interaction
54	path:hsa03030 †	Replication and Repair:DNA replication
55	path:hsa03410 †	Replication and Repair:Base excision repair
56	path:hsa03420 †	Replication and Repair:Nucleotide excision repair
57	path:hsa03430 †	Replication and Repair:Mismatch repair
58	path:hsa03440 †	Replication and Repair:Homologous recombination
59	path:hsa03450	Replication and Repair:Non-homologous end-joining
60	path:hsa03460 †	Replication and Repair:Fanconi anemia pathway
61	path:hsa03060 †	Folding, Sorting and Degradation:Protein export
62	path:hsa04141 †	Folding, Sorting and Degradation:Protein processing in endoplasmic reticulum
63	path:hsa04130 †	Folding, Sorting and Degradation:SNARE interactions in vesicular transport
64	path:hsa04120 †	Folding, Sorting and Degradation:Ubiquitin mediated proteolysis
65	path:hsa04122	Folding, Sorting and Degradation:Sulfur relay system
66	path:hsa03050 †	Folding, Sorting and Degradation:Proteasome
67	path:hsa03018 †	Folding, Sorting and Degradation:RNA degradation
68	path:hsa03010*†	Translation:Ribosome
69	path:hsa00970 †	Translation:Aminoacyl-tRNA biosynthesis
70	path:hsa03013 †	Translation:RNA transport
71	path:hsa03015 †	Translation:mRNA surveillance pathway
72	path:hsa03008 †	Translation:Ribosome biogenesis in eukaryotes
73	path:hsa03020 †	Transcription:RNA polymerase
74	path:hsa03022 †	Transcription:Basal transcription factors
75	path:hsa03040*†	Transcription:Spliceosome

*: significant (fdr<0.05) in pre-ATI   †: significant (fdr<0.05) in post-ATI

Table S2. Selected GO pathways for investigating DALIA-1

	GO ID		Description
1	GO:0002218	†	activation of innate immune response
2	GO:0006956	†	complement activation
3	GO:0002429	†	immune response-activating cell surface receptor signaling pathway
4	GO:0002758	†	innate immune response-activating signal transduction
5	GO:0019883	†	antigen processing and presentation of endogenous antigen
6	GO:0019884	†	antigen processing and presentation of exogenous antigen
7	GO:0048002	†	antigen processing and presentation of peptide antigen
8	GO:0002504	†	antigen processing and presentation of peptide or polysaccharide antigen via MHC class II
9	GO:0002475		antigen processing and presentation via MHC class Ib
10	GO:0002468		dendritic cell antigen processing and presentation
11	GO:0002578		negative regulation of antigen processing and presentation
12	GO:0002579	†	positive regulation of antigen processing and presentation
13	GO:0002577	†	regulation of antigen processing and presentation
14	GO:0002457		T cell antigen processing and presentation
15	GO:0002339		B cell selection
16	GO:0002263	†	cell activation involved in immune response
17	GO:0051607	†	defense response to virus
18	GO:0002432		granuloma formation
19	GO:0002434		immune complex clearance
20	GO:0043299	†	leukocyte degranulation
21	GO:0001909	†	leukocyte mediated cytotoxicity
22	GO:0019724	†	B cell mediated immunity
23	GO:0002228	†	natural killer cell mediated immunity
24	GO:0002707	†	negative regulation of lymphocyte mediated immunity
25	GO:0002708	†	positive regulation of lymphocyte mediated immunity
26	GO:0002706	†	regulation of lymphocyte mediated immunity
27	GO:0002456	†	T cell mediated immunity
28	GO:0002444	†	myeloid leukocyte mediated immunity
29	GO:0002704	†	negative regulation of leukocyte mediated immunity
30	GO:0002705	†	positive regulation of leukocyte mediated immunity
31	GO:0002703	†	regulation of leukocyte mediated immunity
32	GO:0002522		leukocyte migration involved in immune response
33	GO:0002698	†	negative regulation of immune effector process
34	GO:0008228		opsonization
35	GO:0002699	†	positive regulation of immune effector process
36	GO:0002697	†	regulation of immune effector process
37	GO:0002679	†	respiratory burst involved in defense response
38	GO:0002250	†	adaptive immune response
39	GO:0002367	†	cytokine production involved in immune response
40	GO:0006959	†	humoral immune response
41	GO:0002418	†	immune response to tumor cell
42	GO:0002437	†	inflammatory response to antigenic stimulus
43	GO:0006957	†	complement activation, alternative pathway
44	GO:0001867		complement activation, lectin pathway
45	GO:0002227		innate immune response in mucosa
46	GO:0045824	†	negative regulation of innate immune response
47	GO:0045089	†	positive regulation of innate immune response
48	GO:0045088	†	regulation of innate immune response
49	GO:0034341	†	response to interferon-gamma
50	GO:0034340	†	response to type I interferon
51	GO:0050777	†	negative regulation of immune response
52	GO:0002251	†	organ or tissue specific immune response
53	GO:0052555		positive regulation by organism of immune response of other organism involved in symbiotic interaction
54	GO:0002821	†	positive regulation of adaptive immune response
55	GO:0002922		positive regulation of humoral immune response
56	GO:0002839	†	positive regulation of immune response to tumor cell
57	GO:0002863	†	positive regulation of inflammatory response to antigenic stimulus
58	GO:0002830		positive regulation of type 2 immune response
59	GO:0002765	†	immune response-inhibiting signal transduction
60	GO:0002768	†	immune response-regulating cell surface receptor signaling pathway
61	GO:0052552		modulation by organism of immune response of other organism involved in symbiotic interaction
62	GO:0002819	†	regulation of adaptive immune response
63	GO:0002718	†	regulation of cytokine production involved in immune response
64	GO:0043309		regulation of eosinophil degranulation
65	GO:0002920	†	regulation of humoral immune response
66	GO:0002837	†	regulation of immune response to tumor cell
67	GO:0002861	†	regulation of inflammatory response to antigenic stimulus
68	GO:0033006	†	regulation of mast cell activation involved in immune response
69	GO:0043380		regulation of memory T cell differentiation
70	GO:0043313		regulation of neutrophil degranulation
71	GO:0045622	†	regulation of T-helper cell differentiation
72	GO:0002828	†	regulation of type 2 immune response
73	GO:0042092	†	type 2 immune response
74	GO:0002520		immune system development
75	GO:0002366	†	leukocyte activation involved in immune response

76	GO:0050902		leukocyte adhesive activation
77	GO:0042113	†	B cell activation
78	GO:0001767		establishment of lymphocyte polarity
79	GO:0001771		immunological synapse formation
80	GO:0002285	†	lymphocyte activation involved in immune response
81	GO:0030098	†	lymphocyte differentiation
82	GO:0046651	†	lymphocyte proliferation
83	GO:0030101	†	natural killer cell activation
84	GO:0051250	†	negative regulation of lymphocyte activation
85	GO:0031294	†	lymphocyte costimulation
86	GO:0050871	†	positive regulation of B cell activation
87	GO:0045621	†	positive regulation of lymphocyte differentiation
88	GO:0050671	†	positive regulation of lymphocyte proliferation
89	GO:0032816	†	positive regulation of natural killer cell activation
90	GO:0050870	†	positive regulation of T cell activation
91	GO:0050864	†	regulation of B cell activation
92	GO:0045619	†	regulation of lymphocyte differentiation
93	GO:0050670	†	regulation of lymphocyte proliferation
94	GO:0032814	†	regulation of natural killer cell activation
95	GO:0050863	†	regulation of T cell activation
96	GO:0050868	†	negative regulation of T cell activation
97	GO:0046634	†	regulation of alpha-beta T cell activation
98	GO:0046643	†	regulation of gamma-delta T cell activation
99	GO:2001188		regulation of T cell activation via T cell receptor contact with antigen bound to MHC molecule on antigen presenting cell
100	GO:0045580	†	regulation of T cell differentiation
101	GO:0042129	†	regulation of T cell proliferation
102	GO:0046631	†	alpha-beta T cell activation
103	GO:0001768		establishment of T cell polarity
104	GO:0046629	†	gamma-delta T cell activation
105	GO:0035709		memory T cell activation
106	GO:0002286	†	T cell activation involved in immune response
107	GO:0030217	†	T cell differentiation
108	GO:0042098	†	T cell proliferation
109	GO:0002274	†	myeloid leukocyte activation
110	GO:0002695	†	negative regulation of leukocyte activation
111	GO:0002696	†	positive regulation of leukocyte activation
112	GO:0043030	†	regulation of macrophage activation
113	GO:0033003	†	regulation of mast cell activation
114	GO:0030885		regulation of myeloid dendritic cell activation
115	GO:0001776	†	leukocyte homeostasis
116	GO:0050900	†	leukocyte migration
117	GO:0002262	†	myeloid cell homeostasis
118	GO:0002683	†	negative regulation of immune system process
119	GO:0050857	†	positive regulation of antigen receptor-mediated signaling pathway
120	GO:0060369		positive regulation of Fc receptor mediated stimulatory signaling pathway
121	GO:0002253	†	activation of immune response
122	GO:0002687	†	positive regulation of leukocyte migration
123	GO:0070426		positive regulation of nucleotide-binding oligomerization domain containing signaling pathway
124	GO:2000525		positive regulation of T cell costimulation
125	GO:0002645	†	positive regulation of tolerance induction
126	GO:0034123	†	positive regulation of toll-like receptor signaling pathway
127	GO:0002440	†	production of molecular mediator of immune response
128	GO:0002682		regulation of immune system process
129	GO:0002200	†	somatic diversification of immune receptors
130	GO:0045058	†	T cell selection
131	GO:0002507	†	tolerance induction

*: significant (fdr<0.05) in pre-ATI   †: significant (fdr<0.05) in post-ATI
